# Patterns of Mandibular Incisor Agenesis in Japanese Patients With Non-syndromic Oligodontia: A Retrospective Study

**DOI:** 10.7759/cureus.102223

**Published:** 2026-01-24

**Authors:** Dai Ariizumi, Takenobu Ishii, Sayaka Hoshi, Haru Araida, Taiki Morikawa, Yasushi Nishii

**Affiliations:** 1 Department of Orthodontics, Tokyo Dental College, Tokyo, JPN

**Keywords:** canine, congenital missing incisors, japanese population, molar, oligodontia, orthodontic patients

## Abstract

Background

Most studies on congenitally missing teeth include patients with hypodontia because of limited case numbers, whereas reports focusing exclusively on oligodontia are scarce. Although Japanese individuals are reported to have a relatively high prevalence of missing mandibular incisors, the characteristics of mandibular incisor agenesis in Japanese patients with oligodontia and its relationship with overall severity remain unclear.

Methods

This retrospective study included patients diagnosed at the Department of Orthodontics, Tokyo Dental College Chiba Hospital, between 1984 and 2022. Patients with non-syndromic oligodontia, defined as congenital absence of six or more permanent teeth excluding third molars, were identified. The prevalence and distribution of missing mandibular incisors, maxillary and mandibular canines, and molars were analyzed. Associations between the total number of congenitally missing permanent teeth and mandibular incisor involvement were evaluated using Spearman’s rank correlation and categorical analyses.

Results

Among 31,913 orthodontic patients, 228 were diagnosed with oligodontia, representing the largest single-institution cohort of Japanese patients reported to date. The prevalence of oligodontia was 0.7% overall (228/31,913), 0.2% during 1984-2011 (50/22,731), and 1.9% during 2012-2022 (178/9,182), the latter period coinciding with the introduction of national health insurance coverage for orthodontic treatment of oligodontia in Japan. Six missing teeth was the most common presentation, and case numbers decreased with increasing severity. Missing mandibular incisors were observed in 41.2% of patients overall (94/228) and in 93.3% of those with severe oligodontia (15-22 missing teeth) (14/15). Regardless of severity, agenesis of three mandibular incisors was the least common pattern (6-14 missing teeth: 5.0% [4/80]; 15-22 missing teeth: 7.1% [1/14]), whereas agenesis of two incisors was the most frequent (6-14 missing teeth: 50.0% [40/80]; 15-22 missing teeth: 50.0% [7/14]). The proportion of four missing mandibular incisors increased with greater overall severity. A significant positive correlation was identified between the total number of congenitally missing permanent teeth and the number of missing mandibular incisors (Spearman’s r = 0.3961, p < 0.0001). Furthermore, severe mandibular incisor agenesis (3-4 missing incisors) was significantly more prevalent in patients with 15-22 missing teeth than in those with 6-14 missing teeth (40.0% (6/15) vs. 6.1% (13/213), p = 0.0005).

Conclusions

In Japanese patients with oligodontia, mandibular incisors are frequently affected, and both the prevalence and severity of mandibular incisor agenesis increase with the overall severity of congenitally missing teeth. These findings indicate site-specific susceptibility of the mandibular incisor region in patients with extensive oligodontia.

## Introduction

Congenitally missing teeth are a relatively common developmental anomaly. According to the number of missing teeth, this condition is classified into hypodontia (absence of one to five teeth), oligodontia (absence of six or more teeth), and anodontia (complete absence of teeth) [1,2]. The prevalence of congenitally missing permanent teeth varies among ethnic groups, with reported rates of 2.3%-15.7% for hypodontia and 0.08%-1.4% for oligodontia [3-6].

In Japanese populations, most epidemiological studies on congenital tooth agenesis have included hypodontia, whereas investigations restricted to oligodontia remain limited. Although various patterns of missing teeth have been reported in oligodontia [7], previous studies have suggested that mandibular incisor agenesis is relatively common in Asian populations [6-9]. While a high prevalence of missing mandibular incisors has been well documented in hypodontia [8,10,11], data focusing specifically on mandibular incisor agenesis in oligodontia are scarce. Reports on canine agenesis are also limited, although Asians have been reported to exhibit a relatively high prevalence of missing canines [12]. In addition, Japanese individuals have been reported to show a higher frequency of missing canines and second molars compared with other populations [13].

The primary objective of the present study was to clarify the prevalence and severity patterns of mandibular incisor agenesis in Japanese patients with non-syndromic oligodontia, and to examine its association with the total number of congenitally missing permanent teeth. Secondary analyses assessed agenesis of the maxillary and mandibular canines and molars.

## Materials and methods

Medical records of patients who visited the Department of Orthodontics at Tokyo Dental College Chiba Hospital between 1984 and 2022 were retrospectively reviewed. Patients with non-syndromic oligodontia, defined as the congenital absence of six or more permanent teeth excluding third molars, were included. Patients with congenital disorders such as cleft lip and palate and ectodermal dysplasia were excluded.

Data on congenitally missing teeth were extracted based on orthopantomographic findings, with diagnoses confirmed by the attending orthodontist and a senior specialist. Statistical analyses were performed for missing mandibular incisors, maxillary and mandibular canines, and maxillary and mandibular molars.

The number of congenitally missing mandibular permanent incisors (range: 0-4) was treated as count data. To evaluate whether a Poisson regression model was appropriate, the distribution of the outcome was examined. The variance (1.190² = 1.416) exceeded the mean (0.8465), indicating overdispersion. Because overdispersion violates the assumptions of Poisson-based modeling, nonparametric and categorical statistical approaches were adopted.

A monotonic association between the total number of congenitally missing permanent teeth and the number of missing mandibular incisors was assessed using Spearman’s rank correlation coefficient. For categorical analysis, patients were divided into two severity groups based on the total number of congenitally missing permanent teeth, corresponding to absence of up to half (6-14 teeth) or more than half (15-22 teeth) of the 28 permanent teeth excluding third molars, to distinguish moderate and severe oligodontia.

Subsequently, contingency table analysis was performed. When chi-square testing was attempted, the expected cell counts did not satisfy the validity criteria (i.e., all expected counts >1 and ≥80% >5). Therefore, Fisher’s exact test was used to compare the distribution of mandibular incisor agenesis between the two severity groups. All analyses were conducted using GraphPad Prism 9 (GraphPad Software, San Diego, CA, USA), and statistical significance was set at p < 0.05.

This study was approved by the Ethics Committee of Tokyo Dental College (approval number: 1179). The authors declare no conflicts of interest.

## Results

A total of 31,913 patients were diagnosed in the Department of Orthodontics, among whom 228 were diagnosed with oligodontia.

**Figure 1:**
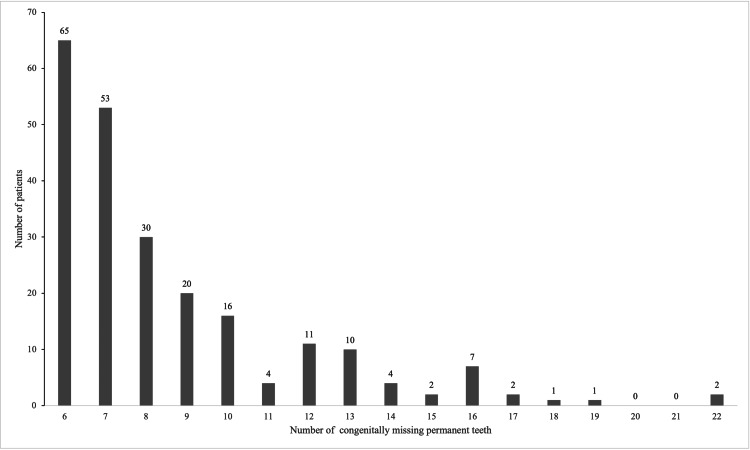
Distribution of the Number of Congenitally Missing Permanent Teeth in Patients with Oligodontia (n=228) The number of patients according to the total number of congenitally missing permanent teeth, excluding third molars. The horizontal axis indicates the number of missing teeth (range: 6–22), and the vertical axis indicates the number of patients. The number of patients decreased as the total number of missing teeth increased.

After orthodontic treatment for oligodontia became covered by the national health insurance system in Japan in 2012, 178 of 9,182 patients were diagnosed with oligodontia, accounting for 78.0% of all oligodontia cases. The prevalence of oligodontia was 0.7% for the entire study period, 0.2% from 1984 to 2011, and 1.9% from 2012 to 2022. The number of missing teeth per patient ranged from six to 22, with patients missing six teeth being the most common. The number of cases decreased as the number of missing teeth increased. No patients with 20 or 21 missing teeth were identified in this cohort (Figure 1). Two patients exhibited the maximum number of missing teeth (22 teeth), and in both cases, the remaining teeth were the maxillary bilateral central incisors and four first molars.

Patients missing 6-10 teeth accounted for approximately 80% of the total cohort. When patients were divided into those missing up to half of the permanent dentition (6-14 teeth) and those missing more than half (15 or more teeth) of the 28 permanent teeth excluding third molars, more than 90% belonged to the 6-14 missing teeth group, whereas fewer than 10% had 15 or more missing teeth. The prevalence of missing canines and second molars increased with the total number of missing teeth. When comparing patients missing up to 14 teeth with those missing 15 or more teeth, the prevalence of agenesis was approximately twofold higher for maxillary canines, fivefold higher for mandibular canines, threefold higher for maxillary second molars, and 1.5-fold higher for mandibular second molars. Overall, agenesis of maxillary canines and maxillary second molars was relatively common, occurring in approximately 30% of cases. Mandibular canine agenesis was relatively uncommon (8.8%), whereas mandibular second molar agenesis showed a consistently high prevalence regardless of the total number of missing teeth (Table 1).

**Table 1:** Patient Distribution and Prevalence of Canine and Second Molar Agenesis According to the Number of Missing Teeth Number and proportion of patients according to the total number of congenitally missing permanent teeth (6–22). The table shows the percentage of patients with at least one missing maxillary canine (U3), maxillary second molar (U7), mandibular canine (L3), and mandibular second molar (L7). Grouped categories include 6–14 (patients with 6–14 missing teeth), 15–22 (patients with 15–22 missing teeth), and all (all patients).

Number of congenitally missing teeth	Number of patients, n	Proportion of patients (%)	Percentage of missing U3 (%)	Percentage of missing U7 (%)	Percentage of missing L3 (%)	Percentage of missing L7 (%)
6	65	28.5	18.5	18.5	3.1	10.8
7	53	23.2	24.5	17.0	1.9	11.3
8	30	13.2	20.0	16.7	6.7	6.7
9	20	8.8	30.0	65.0	5.0	25.0
10	16	7.0	50.0	37.5	18.8	25.0
11	4	1.8	25.0	75.0	25.0	75.0
12	11	4.8	36.4	63.6	18.2	63.6
13	10	4.4	60.0	80.0	20.0	60.0
14	4	1.8	75.0	100.0	25.0	75.0
15	2	0.9	50.0	100.0	0.0	50.0
16	7	3.1	42.9	100.0	14.3	71.4
17	2	0.9	50.0	100.0	50.0	50.0
18	1	0.4	100.0	100.0	0.0	100.0
19	1	0.4	100.0	0.0	100.0	100.0
20	0	0.0	0.0	0.0	0.0	0.0
21	0	0.0	0.0	0.0	0.0	0.0
22	2	0.9	100.0	100.0	100.0	100.0
6-14	213	93.4	27.7	31.5	7.0	62.8
15-22	15	6.6	60.0	93.3	33.3	90.9
All	228	100.0	29.8	35.5	8.8	68.5

The proportions of bilateral agenesis were 75.0% for maxillary canines (all), 65.0% for mandibular canines, 74.1% for maxillary second molars, and 68.5% for mandibular second molars, with similar trends observed across severity groups.

With respect to first molars, the prevalence of agenesis increased with the total number of missing teeth in both the maxilla and mandible; however, neither of the two patients with 22 missing teeth exhibited first molar agenesis. The overall prevalence of missing first molars was 11.8% in the maxilla and 9.2% in the mandible (Table 2).

**Table 2:** Prevalence of Mandibular Incisor and First Molar Agenesis According to the Number of Missing Teeth Number of patients according to the total number of congenitally missing permanent teeth (6–22). The table presents the number and percentage of patients with at least one missing mandibular incisor (L1, L2), as well as the percentage of patients with at least one missing maxillary first molar (U6) and mandibular first molar (L6). Grouped categories include 6–14 (patients with 6–14 missing teeth), 15–22 (patients with 15–22 missing teeth), and all (all patients).

Number of congenitally missing teeth	Number of patients, n	Number of patients with missing mandibular incisors, n	Percentage of missing L1 or / and L2 (%)	Percentage of missing U6 (%)	Percentage of missing L6 (%)
6	65	17	26.2	3.1	0.0
7	53	14	26.4	3.8	3.8
8	30	9	30.0	6.7	0.0
9	20	12	60.0	10.0	15.0
10	16	6	37.5	12.5	6.3
11	4	4	100.0	50.0	0.0
12	11	8	72.7	27.3	18.2
13	10	7	70.0	50.0	40.0
14	4	3	75.0	25.0	25.0
15	2	1	50.0	50.0	100.0
16	7	7	100.0	42.9	57.1
17	2	2	100.0	50.0	50.0
18	1	1	100.0	0.0	0.0
19	1	1	100.0	100.0	100.0
20	0	0	0.0	0.0	0.0
21	0	0	0.0	0.0	0.0
22	2	2	100.0	0.0	0.0
6-14	213	80	37.6	9.9	6.1
15-22	15	14	93.3	40.0	53.3
All	228	94	41.2	11.8	9.2

**Figure 2:**
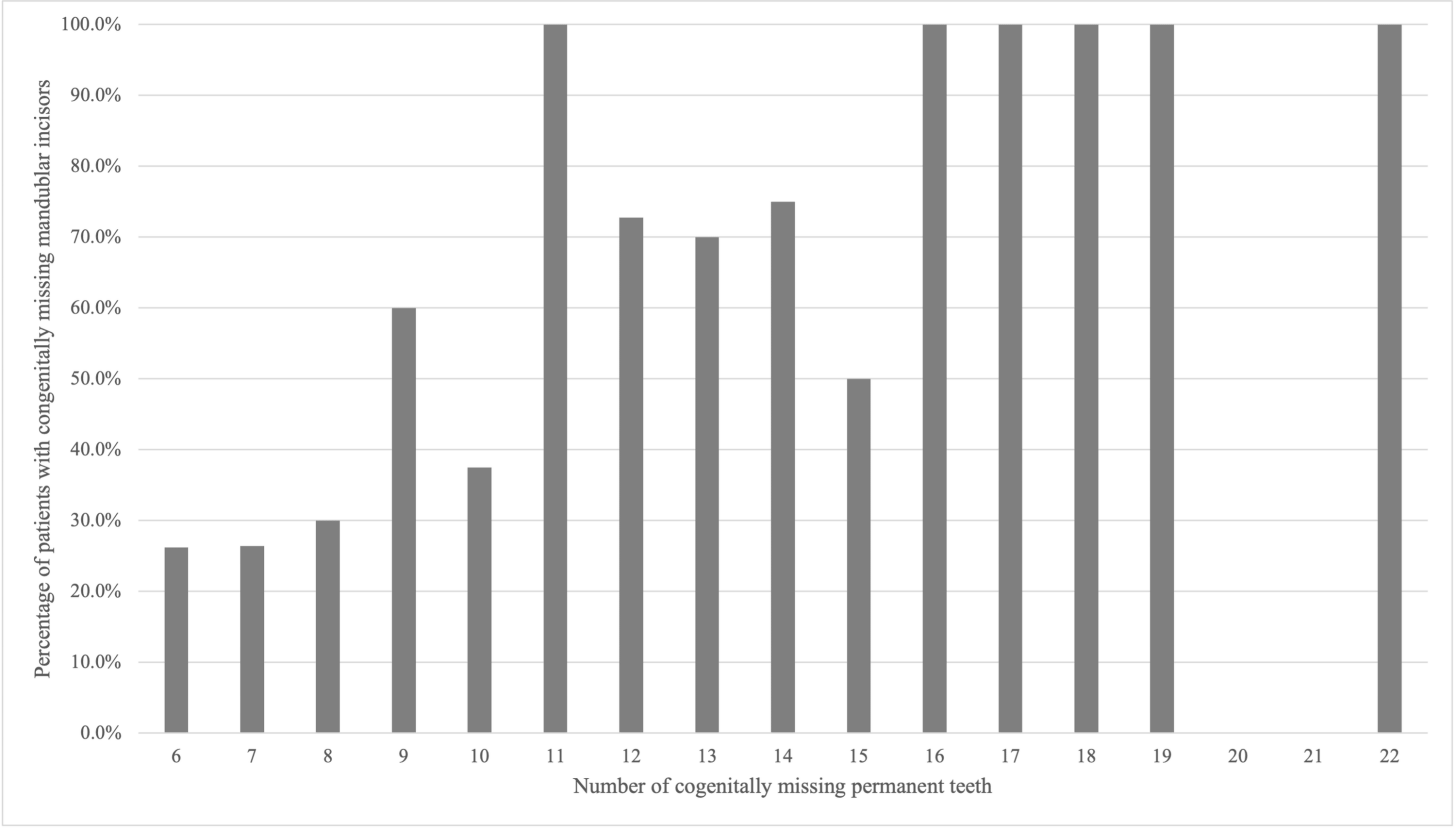
Prevalence of Mandibular Incisor Agenesis in Patients with Oligodontia (n=228) The proportion of patients with at least one congenitally missing mandibular incisor according to the total number of missing permanent teeth. The horizontal axis represents the number of missing teeth (range: 6–22), and the vertical axis represents the percentage of patients with mandibular incisor agenesis. Mandibular incisor agenesis was observed in approximately 50% of patients with 11 or more missing teeth and in all patients with 16 or more missing teeth.

**Figure 3:**
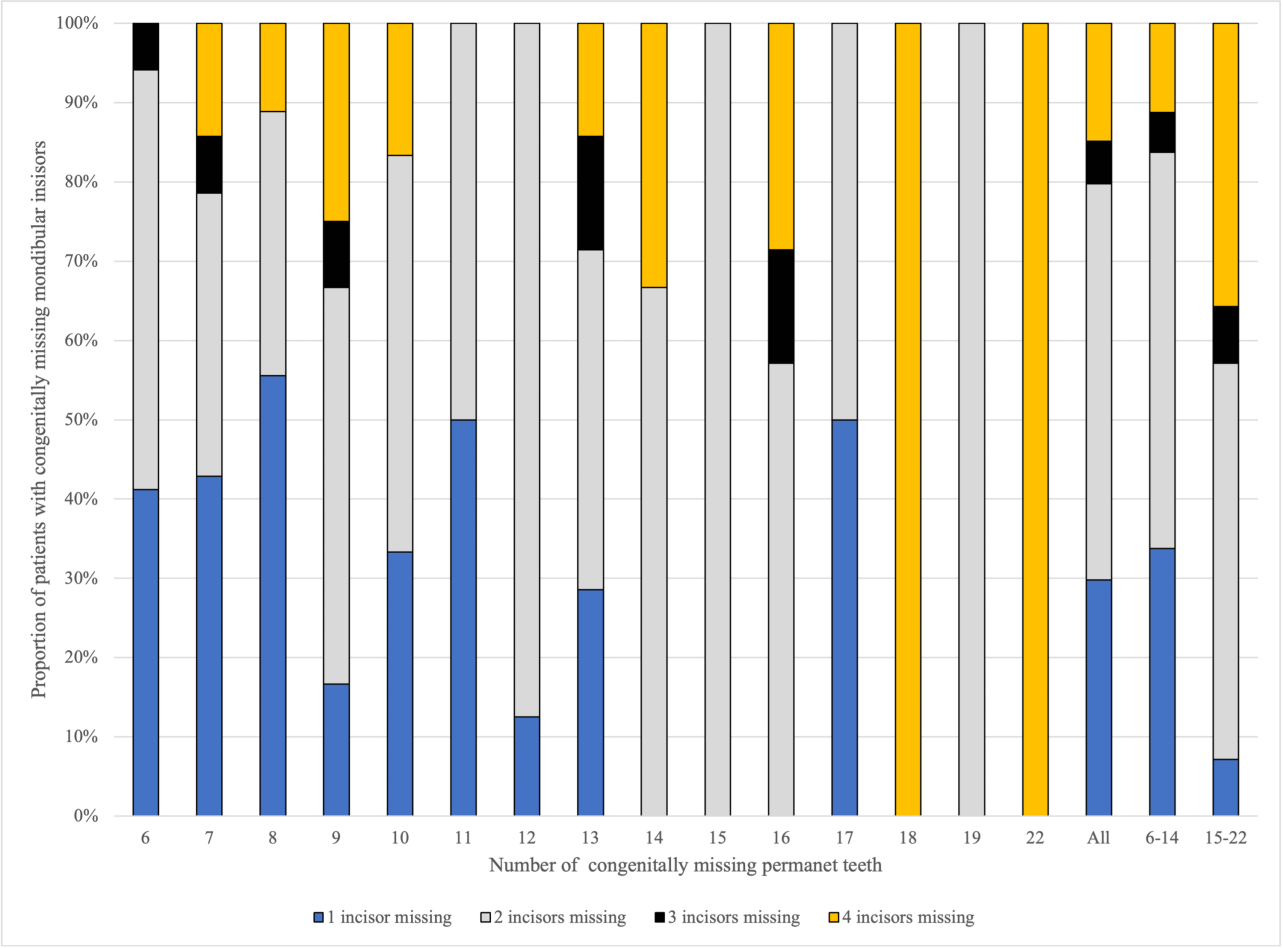
Distribution of the Number of Congenitally Missing Mandibular Incisors in Patients with Oligodontia (n=94) A 100% stacked bar chart showing the distribution of the number of missing mandibular permanent incisors. The horizontal axis represents the total number of missing teeth (6–22), as well as grouped categories (All: all patients; 6–14: patients with 6–14 missing teeth; 15–22: patients with 15–22 missing teeth). The vertical axis indicates the proportion of patients with mandibular incisor agenesis in each group. Blue indicates one missing incisor, gray indicates two missing incisors, black indicates three missing incisors, and yellow indicates four missing incisors.

Regarding mandibular incisors, the proportion of patients missing at least one mandibular incisor was 41.2% overall, 37.6% in the 6-14 missing teeth group, and 93.3% in the 15-22 missing teeth group (Table 2). As observed for other tooth types, the prevalence increased with the total number of missing teeth (Figure 2). In patients with fewer missing teeth, agenesis of a single mandibular incisor was most common, whereas agenesis of all four mandibular incisors was more frequent in patients with more severe oligodontia. Agenesis of two mandibular incisors accounted for approximately half of the cases regardless of severity, whereas agenesis of three mandibular incisors was the least frequent pattern (Figure 3). Among patients with missing mandibular incisors, bilateral agenesis of the corresponding teeth was observed in 64.9%.

**Figure 4:**
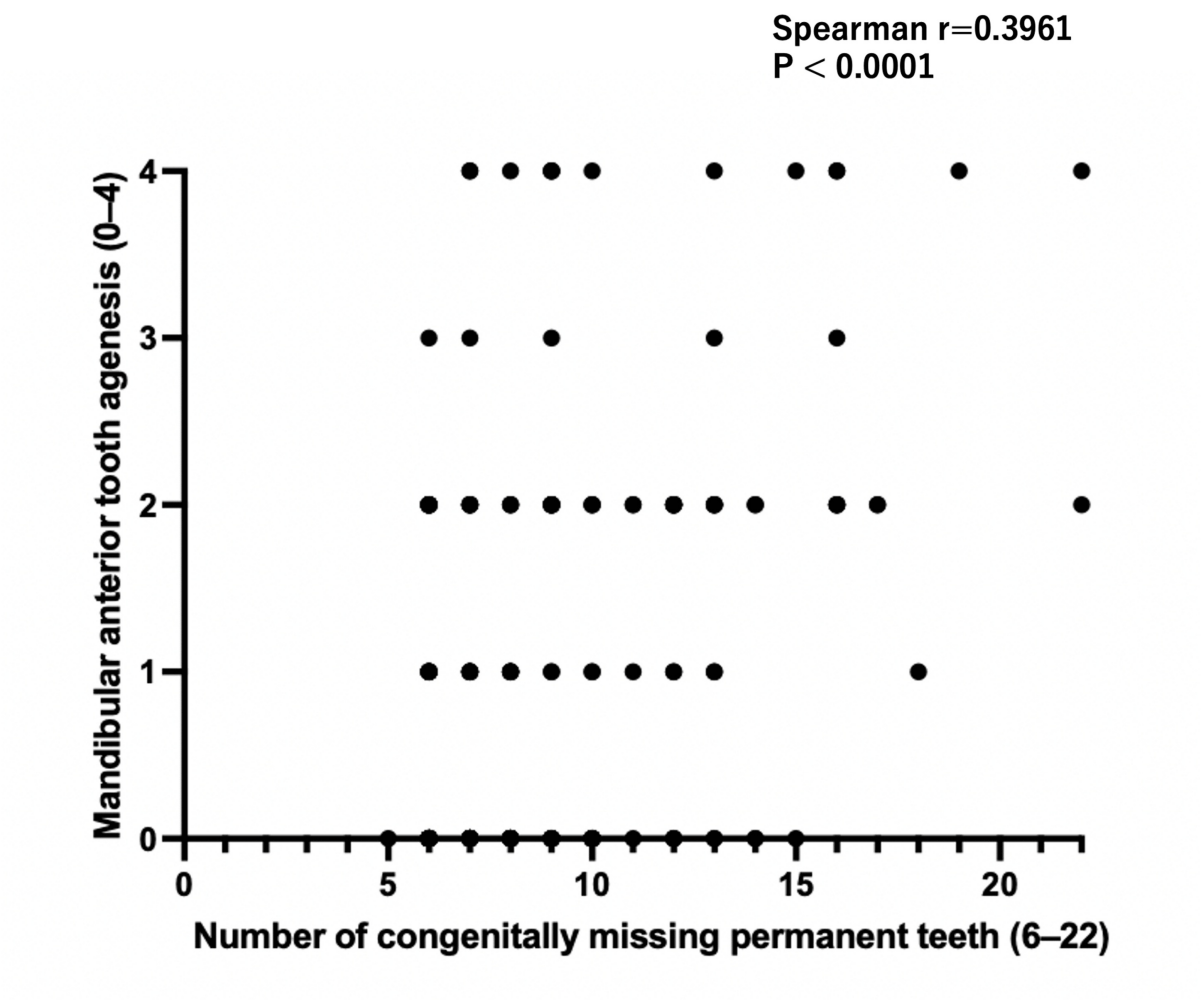
Spearman Correlation Between Total Oligodontia and Mandibular Incisor Agenesis A scatter plot illustrates the monotonic association between the total number of congenitally missing permanent teeth (6–22) and the number of mandibular incisors missing (0–4) in 228 patients.

Spearman’s rank correlation analysis (Figure 4) demonstrated a significant positive association between the total number of congenitally missing permanent teeth and the number of missing mandibular permanent incisors (Spearman’s r = 0.3961; 95% CI, 0.2768-0.5034; p < 0.0001; n = 227). This indicates that patients with a greater number of congenitally missing permanent teeth tend to exhibit a higher number of missing mandibular incisors.

**Figure 5:**
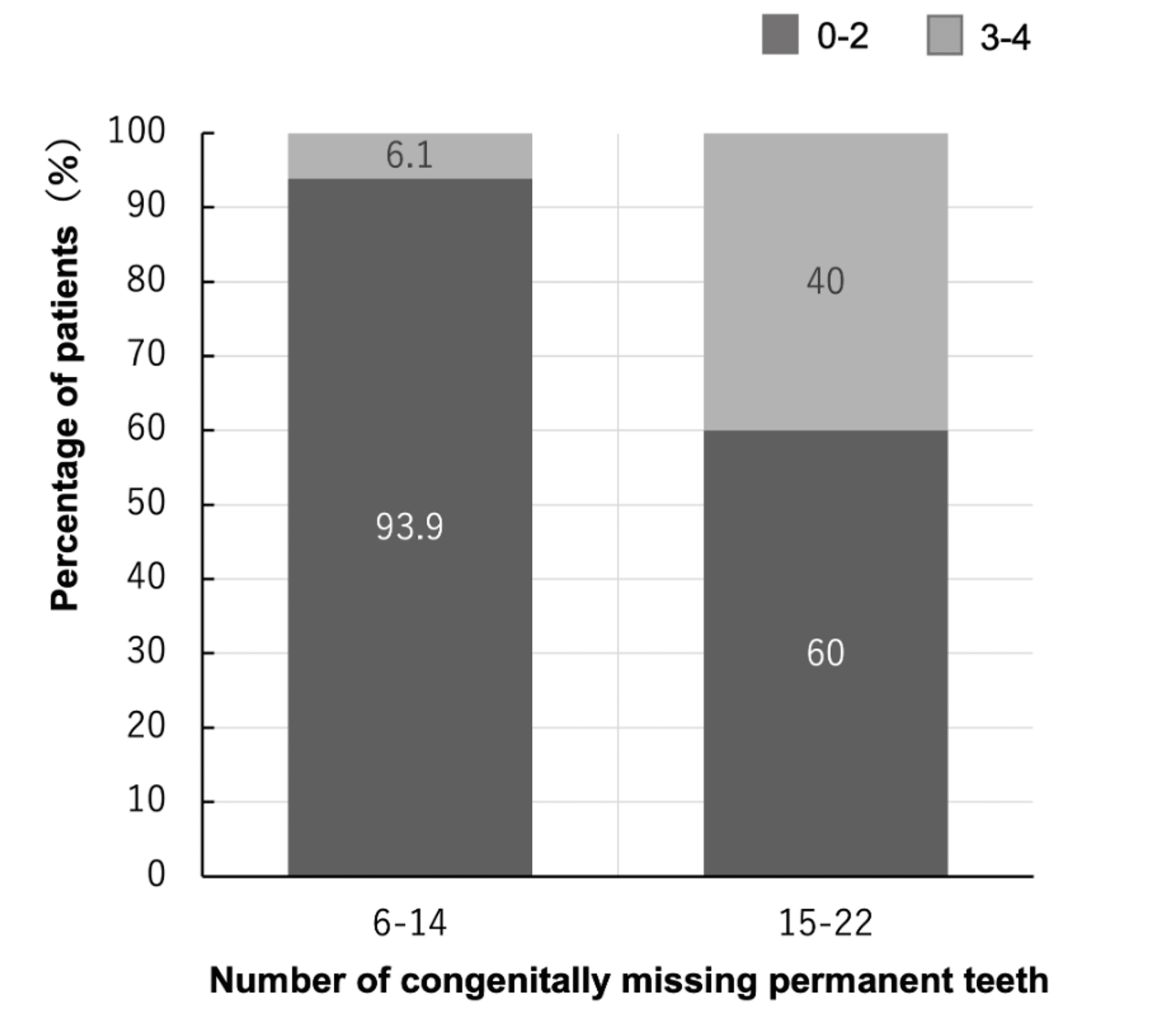
Distribution of Mandibular Incisor Agenesis According to the Severity of Oligodontia (n = 228) A 100% stacked bar chart illustrates the distribution of mandibular incisor agenesis in Japanese patients with non-syndromic oligodontia, stratified by overall severity: 6–14 congenitally missing teeth (n = 213) and 15–22 congenitally missing teeth (n = 15). In the 6–14 missing teeth group, 0–2 missing mandibular incisors were observed in 200 patients (93.9%), whereas 3–4 missing mandibular incisors were observed in 13 patients (6.1%). In the 15–22 missing teeth group, 0–2 missing mandibular incisors were observed in nine patients (60.0%), and 3–4 missing mandibular incisors were observed in six patients (40.0%).

In the contingency analysis, severe mandibular incisor agenesis (3-4 missing incisors) was observed in 6.1% of patients with 6-14 missing permanent teeth (13/213), but in 40.0% of those with 15-22 missing teeth (6/15) (Figure 5). Fisher’s exact test (Table 3) showed that this difference was statistically significant (p = 0.0005). Thus, patients with a larger number of congenitally missing permanent teeth were substantially more likely to present with severe mandibular incisor agenesis.

**Table 3:** Fisher’s Exact Test for the Association Between Oligodontia Severity and Mandibular Incisor Agenesis A 2 × 2 contingency table comparing the prevalence of mandibular incisor agenesis (0–2 vs. 3–4 missing teeth) between the mild oligodontia group (6–14 congenitally missing permanent teeth) and the severe oligodontia group (15–22 missing permanent teeth). Fisher’s exact test was used; p = 0.0005.

Total missing teeth	Mandibular incisors 1-2	Mandibular incisors 3-4	Total
6-14	200	13	213
15-22	9	6	15
Total	209	19	228

## Discussion

In the present study, 228 patients with oligodontia were included, representing the largest single-institution dataset of Japanese patients with oligodontia reported to date. The prevalence of oligodontia differed by approximately tenfold before and after orthodontic treatment became covered by national health insurance in Japan. Because 78.0% of all oligodontia patients were identified after 2012, these findings suggest that awareness of oligodontia has increased substantially in Japan since that time. The prevalence of 1.9% from 2012 to 2022 was comparable to that reported in a previous Japanese study (prevalence of 1.4% in general: 1.8% for girls and 0.9% for boys during 1995-2007) [6]. Consistent with previous reports [6,7], cases with six missing teeth were the most common among patients with oligodontia.

Regarding canine agenesis, Sivarajan et al. [12] reported that the global population prevalence of canine agenesis was 0.30% (0.0-4.7%), highest in Asia (0.54%). Canine agenesis was more common in the maxilla (88.57%), followed by both maxilla and mandible (8.57%), and the least common was mandible-only presentation (2.86%). In Asia, unilateral agenesis was almost twice as prevalent as bilateral, but in Europe, the bilateral form was more common. The prevalence of canine agenesis in the maxilla (between 0.03% and 4.7%) was higher than that in the mandible (between 0% and 1.12%)*. *In contrast, our results demonstrated that the prevalence of missing maxillary canines was 29.8%, whereas that of mandibular canines was 8.8%. Bilateral agenesis was more common than unilateral agenesis for both maxillary (75.0%) and mandibular canines (65.0%), differing from the findings reported by Sivarajan et al. Comparison of the 6-14 and 15-22 missing teeth groups suggested that mandibular second molars, maxillary canines, maxillary second molars, and mandibular canines were affected in that order with respect to the influence of the total number of missing teeth.

With respect to first molars, the finding that both patients with 22 missing teeth retained all first molars is consistent with previous reports indicating that first molars are among the least likely teeth to be congenitally missing [6,14,15]. However, not all oligodontia patients retained first molars, as first molar agenesis was observed in 11.8% of cases in the maxilla and 9.2% in the mandible. This discrepancy from the findings of Goya et al. [6], who reported that *no first molars were missing in any case*, is likely attributable to differences in study populations, as their cohort consisted primarily of patients with hypodontia rather than oligodontia.

Regarding missing mandibular incisors, agenesis of three incisors was the least frequent pattern regardless of severity, whereas agenesis of two incisors was the most common. As severity increased, the proportion of patients missing all four mandibular incisors also increased. Whether this pattern is specific to Japanese patients with oligodontia remains unclear, as comparable studies focusing on mandibular incisors in single-ethnicity oligodontia cohorts are lacking. Future studies in other populations are warranted.

The present findings demonstrate a clear, monotonic relationship between overall congenital tooth agenesis severity and mandibular incisor involvement. The positive Spearman correlation indicates that as the total number of congenitally missing permanent teeth increases, mandibular incisor agenesis becomes more prevalent. Moreover, categorical analysis revealed a markedly higher proportion of severe mandibular incisor agenesis (3-4 missing incisors) in patients with 15-22 missing permanent teeth compared with those missing 6-14 teeth. These results support the concept of site-specificity within the dentition, suggesting that the mandibular incisor region is particularly susceptible in patients with extensive congenital tooth agenesis. This pattern may reflect shared developmental pathways or regional genetic sensitivity affecting anterior mandibular morphogenesis.

As the number of missing teeth increases, the number of remaining teeth decreases, potentially reducing the need for orthodontic treatment alone. Consequently, it is difficult for orthodontic departments to accumulate sufficient numbers of patients with nearly 20 missing teeth. To comprehensively characterize oligodontia, data from pediatric dentistry and prosthodontic departments are also required.

This study has several limitations. First, the study population was limited to patients with oligodontia who sought orthodontic treatment. Patients who did not require orthodontic intervention, such as those with healthy retained primary teeth or those requiring prosthetic treatment alone due to extensive tooth agenesis, were not included. Therefore, the prevalence of mandibular incisor agenesis may differ if such cases are taken into account.

Second, data regarding missing tooth types were collected based on orthopantomographic records at the initial diagnosis. Consequently, cases with extremely delayed development of the second molar tooth germ, as well as cases in which the second molar was absent but the third molar erupted as a substitute, may have been included. As a result, the reported prevalence of second molar agenesis may lack complete accuracy.

In addition, temporal confounding cannot be fully excluded, as changes in diagnostic awareness and health insurance coverage over the long study period may have influenced case identification. Although diagnoses were confirmed by both attending orthodontists and senior specialists, formal examiner calibration was not performed, and examiner reliability could not be quantitatively assessed. Furthermore, this study was not designed to investigate etiologic mechanisms, and genetic or developmental factors underlying mandibular incisor agenesis could not be directly evaluated.

Finally, this was a single-institution study involving only Japanese patients, and no direct comparisons with other countries were performed. Therefore, it remains unclear whether the findings observed in this study are specific to Japanese patients with oligodontia or represent characteristics shared by Asian populations in general. Further large-scale, multicenter studies are warranted to clarify these issues.

## Conclusions

In Japanese patients with nonsyndromic oligodontia, mandibular incisors were frequently affected by congenital tooth agenesis. The likelihood and extent of mandibular incisor agenesis increased as the overall number of missing permanent teeth increased, indicating a severity-dependent pattern. These findings suggest a site-specific vulnerability of the mandibular incisor region in patients with multiple congenitally missing teeth. Recognition of this tendency may be clinically relevant for diagnosis, treatment planning, and long-term management of oligodontia.
